# ASO Author Reflections: Realtime Near-infrared Autofluorescence Based Overlay Imaging as Intraoperative Tool for Parathyroid Gland Localization: Routinized Application and Standards in Clinical Practice

**DOI:** 10.1245/s10434-023-14701-y

**Published:** 2023-12-12

**Authors:** Melisa Arikan, Josef Hegazy, Sophie Mertlitsch, Teresa Binter, Lindsay Hargitai, Christian Scheuba, Philipp Riss

**Affiliations:** Division of Visceral Surgery, Department of General Surgery, Medical University of Vienna, Vienna, Austria

## Past

Over the past years, near-infrared (autofluorescence) imaging has been increasingly used intraoperatively and further developed to visualize parathyroid glands (PGs) to reduce the risk of postoperative hypoparathyroidism after endocrine neck surgery.^1^ Elevision IR (Medtronic, Dublin, Ireland) is a development of near-infrared autofluorescence (NIRAF) imaging, which differs from current products in its (threshold)overlay imaging resulting from white- and near-infrared (NIRF) light overlapping. Furthermore, an intraoperative real-time image is generated on a display monitor. Application of overlay imaging using Elevision IR in clinical practice was first introduced in a case report of ICG-guided parathyroidectomy, followed by preliminary results using NIRAF based overlay imaging on 25 patients.^2,3^ Yet, to date no standards for the use of NIRAF-based overlay imaging for intraoperative PG detection have been defined.

## Present

This prospective study aimed to define standards for the usage of Elevision IR in endocrine neck surgery and to demonstrate its clinical feasibility.^4^ The findings are important to faciliate the application of NIRAF based overlay imaging. Patients who had undergone thyroid and/or parathyroid surgery and in whom Elevision IR was applied to visualize at least one parathyroid gland were included in this study.

Reduction in time needed for the application of the device was achieved as experience was gained. A moderate degree of accordance for the detection of PGs between the surgeon and overlay imaging was described. However, in some cases Elevision IR could visualize even more PGs, especially on the specimen, which was subsequently scanned after thyroidectomy. Ideal application time, distance, infrared intensity, and angle for each measurement were defined. Various NIRAF patterns and intensities were described in a patient collective with a large number of mixed pathologies.

## Future

NIRAF-based overlay imaging has been shown to be a good and easily applied adjunct tool in clinical practice. Apart from the capability of detecting PGs, NIRAF based overlay imaging can provide us with new information about PGs through the variety of autofluorescence patterns in terms of different pathologies. To extend and specify our standards for subgroups of patients and to analyze the mechanisms for these various patterns, further prospective studies are necessary. Those can serve as basis for standard-operating procedures on the use of NIRAF-based overlay imaging in thyroid and parathyroid surgeries. Furthermore, defining standards for the routinized use of indocyanine green in endocrine neck surgery are pending. The introduction of guidelines for the use of this new technique are of high necessity.
